# Platelet P2Y12 Inhibitor in the Treatment and Prevention of Migraine: A Systematic Review and Meta-Analysis

**DOI:** 10.1155/2022/2118740

**Published:** 2022-03-20

**Authors:** Fengzhi Wang, Yumeng Cao, Yanjie Liu, Zhanxiu Ren, Fuyong Li

**Affiliations:** ^1^Department of Neurology, People's Hospital of Liaoning Province, People's Hospital of China Medical University, Shenyang, China; ^2^Department of Neurology, Baoji Central Hospital, Baoji, China; ^3^Department of Clinical Laboratory, People's Hospital of Liaoning Province, Shenyang, China; ^4^Department of Neurosurgery, People's Hospital of Liaoning Province, People's Hospital of China Medical University, 33 Wenyi Road, Shenhe District, Shenyang 110016, China

## Abstract

There have been speculation and research linking migraine with abnormalities of platelet aggregation and activation. The role of the P2Y12 platelet inhibitor in the treatment of migraine has not been established. We aim to evaluate the efficacy of the platelet P2Y12 inhibitor in the treatment of migraine and prevention of new-onset migraine headache (MHA) following transcatheter atrial septal defect closure (ASDC). We searched the PubMed, Web of Science, and Cochrane Library databases for relevant studies. The primary outcomes were the headache responder rate and the rate of new-onset migraine attacks following ASDC. Four studies for a total of 262 migraine patients with or without patent foramen ovale (PFO) and three studies involving 539 patients with antiplatelet treatment in the prevention of new-onset migraine following ASDC were included. The pooled responder rate of the P2Y12 inhibitor for migraine was 0.64 (95% CI: 0.43 to 0.81). For patients who underwent ASDC, the use of antiplatelet regimens including the P2Y12 inhibitor, compared with regimens excluding P2Y12 inhibitor, resulted in a lower rate of new-onset migraine (OR: 0.41, 95% CI: 0.22 to 0.77, *P* = 0.005). We concluded that the P2Y12 platelet inhibitor may have a primary prophylactic role in migraine patients with or without PFO and prevent new-onset MHA after ASDC. The responsiveness of the P2Y12 inhibitor could help select candidates who would benefit from PFO closure. It warrants further large-scale research to explore the role of the P2Y12 inhibitor, particularly in a proportion of migraine patients.

## 1. Introduction

Migraine is a recurrent, disabling neurological disorder with an estimated 1-year prevalence of 12% in the general population [1]. The World Health Organization ranks migraine as the third most common disorder and the second most disabling neurological disorder in the world [2, 3]. The pathogenesis of migraine is complex and multifaceted and, thus far, has not been elaborated clearly. There have been speculation and research linking migraine with abnormalities of platelet morphology or function [4]. Early theories centered on the neurotransmitter serotonin, which plays a significant role in the pathophysiology of migraine. Platelets carry large amounts of serotonin within the circulation and share many structural, biochemical, and pharmacological properties with serotonergic nerve endings [5]. Currently, the prevailing hypothesis is that migraine should be considered a primary neurovascular disorder, triggered by a relative reduction of cerebral blood flow in the affected cerebral area. Mounting epidemiological evidence have demonstrated that migraine, particularly migraine with aura, is an independent risk factor for cerebral ischemia [6], which may also imply the role of platelets in a proportion of migraine patients. There is also a biologically plausible link between platelet aggregation and patent foramen ovale (PFO) and migraine or migraine headaches after transcatheter atrial septal defect closure (ASDC) [7, 8]. A better understanding of the possible contribution of platelets in migraine might be valuable for the treatment and prevention of migraine in at least a subset of patients.

Recent evidence suggested that the P2Y12 inhibitor, targeting platelet adenosine diphosphate P2Y12 receptors and specifically inhibiting adenosine diphosphate-stimulated platelet function, may be effective in treating migraine with or without PFO and preventing new-onset migraine following transcatheter ASDC [8–15]. At present, there are two broad categories of approved P2Y12 platelet receptor inhibitors: irreversible thienopyridines and reversible ATP analogs. Thienopyridines include ticlopidine, clopidogrel, and prasugrel; ATP analogs are ticagrelor and cangrelor. In view of the potential link between platelet and migraine, we perform a meta-analysis to evaluate the efficacy of the platelet P2Y12 inhibitor in the treatment of migraine and prevention of the occurrence and reduction of the number of new-onset migraine headache (MHA) following ASDC.

## 2. Methods

### 2.1. Literature Search

This systematic review was conducted in accordance with PRISMA (Preferred Reporting Items for Systematic Reviews and Meta-Analyses) guidelines [16] and Cochrane Handbook for Systematic Reviews [17]. Two investigators independently searched the PubMed, Cochrane Library, and Web of Science databases, with no language restrictions, for articles published from the inception to May 2020. They also manually searched the reference lists of relevant articles. The search strategy was as follows:

1. “ticlopidine” or “clopidogrel” or “prasugrel” or “ticagrelor” or “cangrelor” or “thienopyridine” or “P2Y12 inhibitor” or “anti-platelet”
2. “migraine” or “cephalgia” or “cephalalgia” or “headache”
3. 1 and 2

### 2.2. Study Selection

Two clinical research fellows reviewed each study for eligibility. Reviews, meeting abstracts, comments, letters, animal studies, and duplicate studies were removed from the retrieved records. The remaining articles were screened based on their titles and abstracts, and the papers thus selected then underwent full-text review. All disagreements were resolved by consensus with the help of a third reviewer. We included published studies fulfilling the following criteria: (1) all of the identified clinical trials of patients who received P2Y12 inhibitor for migraine with or without PFO; (2) trials compared the efficacy of antiplatelet regimens including the P2Y12 inhibitor to other antiplatelet regimens excluding the P2Y12 inhibitor for the prevention of new-onset migraine attacks following ASDC; (3) migraine diagnosed based on the diagnostic criteria of the International Headache Society (IHS) or confirmed by certificated neurologists; and (4) trials that reported efficacy outcome(s) with data that could be extracted. All disagreements were resolved by consensus with the help of a third reviewer.

### 2.3. Outcome Measurement

Outcomes were extracted as dichotomous or continuous variables. The primary outcomes were headache responder rate and rate of new-onset migraine attacks following ASDC. Headache responder was measured differently across the enrolled studies, including (1) those with ≥50% reduction in monthly MHA days compared with baseline [10, 11] and (2) those with >50% reduction or complete elimination of migraine symptoms [12]. The secondary outcome was the rate of migraineurs with the ongoing benefit of the responder who underwent PFO closure, after discontinuation of P2Y12 platelet inhibitors.

### 2.4. Risk-of-Bias Assessment and Publication Bias

Data extraction was independently conducted by two authors. The demographic and baseline characteristics, therapy designs, and efficacy results were extracted from the eligible studies. The quality of the randomized controlled trials (RCTs) was assessed using the modified Jadad scale with a score ranging from 0 to 7 [18]. The scale includes four main parameters: the generation of random sequences, randomized concealment, blinding, and monitoring of subject dropouts. Higher scores suggest higher quality. Trials are considered poor quality if the score ≤ 3. Scoring was completed by two authors, and disagreements were resolved by consensus with the help of a third reviewer. Given the small number of studies included, publication bias could not be effectively presented using a funnel plot; thus, publication bias may exist.

### 2.5. Data Synthesis and Analysis

Statistical analysis was conducted by two authors using the RevMan v5.3 software from the Cochrane Collaboration. Responder rates of the P2Y12 inhibitor for migraine were assessed by meta-analysis using the Metaprop module in the R-3.6.1 statistical software package. The rates reported in each study were logit transformed prior to computing the pooled rate. Continuous variables were expressed as mean ± SD. For dichotomous variables, odds ratios (ORs) and 95% confidence intervals (CIs) were selected. The Cochran *Q* test and *I*^2^ statistic were used to detect heterogeneity. Either *P* < 0.1 or *I*^2^ > 50% was defined as significant heterogeneity. A random-effects model was chosen if significant heterogeneity was present among the studies; otherwise, a fixed-effects model was chosen to calculate pooled estimates. Statistical significance was defined as *P* < 0.05.

## 3. Results

### 3.1. Study Characteristics

**Figure 1:**
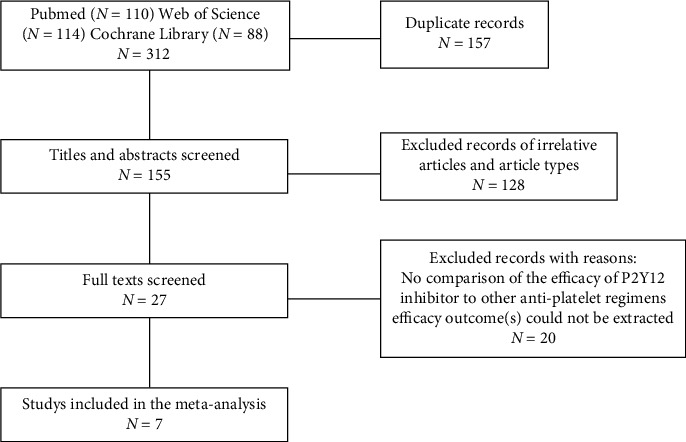
PRISMA flow chart for inclusion of eligible studies.

The search flow diagram is shown in Figure 1. After applying the inclusion criteria, four studies for a total of 262 migraine patients with or without PFO (Table 1) and three studies involving 539 patients with antiplatelet treatment for the prevention of new-onset migraine attacks following ASDC (Table 2) were included. These studies consisted of two RCTs [9, 14], three open-label single-arm feasibility studies [10–12], and two retrospective observational studies [13, 15]. Both of the two RCTs were scored at 7 using the Jadad scoring system (Supplemental Table 1), suggesting high quality. The mean age of the patients was 38.2 years. 543 (67.8%) out of 801 participants were female, and in one trial [12], all of the participants were female. For studies that assessed the P2Y12 inhibitor for migraine with or without PFO, the mean age of the patients was 39.3 years, and 133 (50.8%) out of 262 participants had migraine with aura. For studies that assessed the P2Y12 inhibitor in the prevention of new-onset MHA after transcatheter ASDC, the mean age of the patients was 37.7 years, and one trial [13] also enrolled pediatric patients aged from 4 to 79 years. Among them, the RCT enrolled patients with an indication for ASDC and no history of migraine, while the two retrospective studies enrolled patients with or without migraine.

### 3.2. Efficacy Outcomes

#### 3.2.1. Platelet P2Y12 Inhibitor for Migraine with or without PFO

One RCT [9] investigated patients with migraine, and three open-label single-arm feasibility trials [10–12] assessed patients with MHA and synchronous PFO. In the first and the only RCT, no statistically significant effect of clopidogrel was found as a prophylactic treatment for migraine. In this trial, patients (*n* = 80) were randomized to one of the two groups: clopidogrel 75 mg daily (*n* = 39; the clopidogrel group) or placebo (*n* = 41; the control group) for three months. The number of headache days fell by 1.9 on clopidogrel and 1.8 on placebo (adjusted difference 0.02, CI: 2.07 to 2.12). Headache severity rose by 0.14 points on clopidogrel and fell by 0.63 on placebo (adjusted difference 0.7, CI: 0.11 to 1.57), and the main treatment effect did not depend on the presence or absence of migraine with aura and the presence or absence of a PFO or atrial septal aneurysm.

On the other hand, all of the three open-label single-arm feasibility trials showed a favorable MHA response in patients with PFO using the P2Y12 platelet inhibitor clopidogrel, prasugrel, or ticagrelor. In one trial [11], clopidogrel was offered to patients firstly, and MHA nonresponders with inadequate platelet inhibition were then offered prasugrel. Thienopyridine-responsive patients were then offered PFO closure. Of the 136 patients, 80 were MHA responders to clopidogrel, and 10 were responders to prasugrel. Overall, 90 (66%) patients treated with thienopyridines were MHA responsive. 56 of the 90 responders received PFO closure, and 52 of them had ongoing effective MHA reduction after discontinuation of the thienopyridine (3 months after closure), with a follow-up range of up to 6 years. The other 26 MHA responders continued on thienopyridine therapy, with ongoing headache response for periods as long as 4 years. The clopidogrel responder rate was equivalent in episodic, chronic, aura, and nonaura subgroups. Another trial [10] investigated the effects of ticagrelor, a nonthienopyridine P2Y12 inhibitor, as a prophylactic treatment for refractory migraine/PFO. Overall, 17 participants (43%) were ticagrelor MHA responders. Consistent with the study by Sommer et al. [11], MHA responder rates were not statistically different in participants with episodic or chronic MHA, with or without aura. The other study by Spencer et al. [12] also assessed the effects of clopidogrel added to the existing prophylactic migraine regimen for patients with severe MHA and PFO. 13 (87%) out of the 15 participants had >50% reduction or complete elimination of migraine symptoms, and 8 of the 9 responders underwent PFO closure who had ongoing benefit after the discontinuation of clopidogrel.

**Figure 2:**
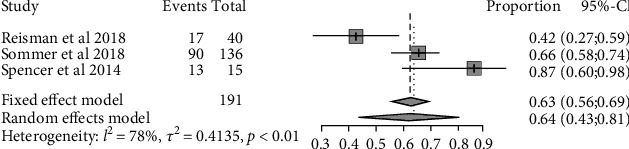
Forest plot of the P2Y12 platelet inhibitor for migraine headaches responder rate in migraine with or without PFO.

**Figure 3:**
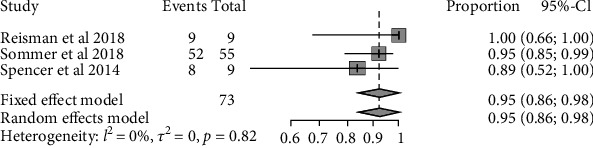
Forest plot of the P2Y12 platelet inhibitor for the rate of migraineurs with the ongoing benefit of the responder who underwent PFO closure, after discontinuation of the P2Y12 platelet inhibitor.

The pooled responder rate of the three trials was 0.64 (95% CI: 0.43 to 0.81) (Figure 2). Of the responders who underwent PFO closure, after P2Y12 platelet inhibitor discontinuation, the pooled rate of migraineurs with ongoing benefit was 0.95 (95% CI: 0.86 to 0.98) (Figure 3).

#### 3.2.2. Platelet P2Y12 Inhibitor for the Prevention of New-Onset MHA after Transcatheter ASDC

Three trials were enrolled, including one RCT [14] and two retrospective studies [13, 15] comparing the efficacy of antiplatelet regimens including P2Y12 inhibitors with other antiplatelet regimens excluding P2Y12 inhibitors in the prevention of new-onset migraine attacks following ASDC. The RCT assessed the efficacy of clopidogrel plus aspirin and aspirin plus placebo in the prevention of new-onset migraine attacks following ASDC. One of the retrospective trials [15] compared the effect of aspirin for six months plus clopidogrel 75 mg daily for the first month with aspirin alone for six months. In the other retrospective trial [13], antiplatelet regimens including P2Y12 inhibitors were clopidogrel, ticlopidine, aspirin plus ticlopidine, and ticlopidine plus warfarin; antiplatelet regimens excluding P2Y12 inhibitors were aspirin, aspirin plus warfarin, and dipyridamole plus warfarin.

**Figure 4:**
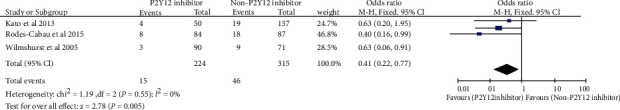
Forest plot of antiplatelet regimens including P2Y12 inhibitors vs. antiplatelet regimens excluding P2Y12 inhibitors in preventing new-onset migraine headaches after transcatheter ASDC.

For patients who underwent ASDC, the use of antiplatelet regimens including P2Y12 inhibitors, compared with regimens excluding P2Y12 inhibitors, resulted in a lower rate of new-onset migraine (OR: 0.41, 95% CI: 0.22 to 0.77, *P* = 0.005) (Figure 4). No heterogeneity was found among the studies for this analysis (*I*^2^ = 0, *P* = 0.55).

## 4. Discussion

There is an ongoing search for new treatments for patients suffering from migraine, and it is especially important to choose appropriate drugs tailored to particular migraine populations. Treatment with monoclonal antibodies against calcitonin gene-related peptide (CGRP) is a new generation of neurobiology-related and mechanism-based preventive antimigraine therapy. As is known to all, CGRP is a proinflammatory and vasoactive neuropeptide involved in peripheral and central sensitization in the pathophysiology of migraine. As the first fully human monoclonal antibody against the CGRP receptor, erenumab is an efficacious and well-tolerated preventive treatment in migraineurs [19], but study shows that there were no effects of erenumab on platelets in vitro (by binding, activation, or phagocytosis assays) [20], and there is also no subgroup study analyzing the prophylactic efficacy of CGRP receptor antagonists in migraine patients with PFO. The pathogenesis of migraine is complex and multifaceted, and in this meta-analysis, we focused on the “platelet aggregation and activation” of migraine and aimed to provide better treatment options for particular migraine populations (such as migraineurs with PFO).

In this systematic review and meta-analysis, we enrolled four studies for a total of 262 migraine patients with or without PFO, and three studies involved 539 patients for the prevention of new-onset migraine attacks following ASDC with the antiplatelet treatment of the platelet P2Y12 inhibitor. Among them, only two RCTs were identified, and others were all observational studies; thus, we could not draw a definite conclusion from these data. Overall, in the present review, the favorable effects of antiplatelet medication of the P2Y12 inhibitor for migraine, the nearly parallel (95%) MHA response to subsequent PFO closure, and the lower rate of new-onset migraine after ASDC all suggest a platelet-mediated or platelet-activated mechanism of migraine. The P2Y12 platelet inhibitor may have a primary prophylactic role in migraine patients with or without PFO and prevent new-onset MHA after ASDC. What is more, the responsiveness of the P2Y12 inhibitor could help select candidates who would benefit from PFO closure.

A study of the relationship between migraine headache and hematological parameters has shown that the platelet/lymphocyte ratio, mean platelet volume, and platelet values were higher in migraine patients compared to the control group [21]. Increased platelet activity during or between migraine attacks has been early reported [22–25]. Activation and aggregation of platelets may be initiated by certain triggers (e.g., estrogens [26], cold weather [27], emotional and physical stress [28], and additives in food and beverages [29]) or factors (e.g., shear stress), which increase platelet intracellular calcium levels. This leads to the release of serotonin and adenosine diphosphate from the platelet, which reinforces the aggregation. Platelet activation evoked by elevated shear stress and release of serotonin from platelets may be important in a migraine attack, especially in patients with PFO [30]. There is evidence that serotonin metabolism is impaired in migraineurs [24, 31]. A chronic low serotonin disposition in the brain has been recognized as one of the leading biochemical pathogenesis of migraine, and a sudden increase of serotonin represents a crucial trigger of migraine attacks [31]. A cohort study investigating the burden of migraine and levels of serotonin in adults with an atrial septal defect found that patients with atrial septal defect had an increased risk of receiving a migraine diagnosis (hazard ratio: 3.4, 95% CI: 2.6 to 4.6) and plasma serotonin was severely elevated [32]. Besides, a model of endothelial dysfunction in the trigeminovascular system has emerged in migraine research recently, which induces an inflammatory response and a hypercoagulable state marked by increased platelet reactivity, altered erythrocyte morphology and metabolism, and increased fibrinogen levels [33, 34]. In addition, hypotension, possibly part of the autonomic dysfunction that causes nausea and emesis, has been observed in a number of patients during migraine attacks [35]. Blockade of platelet adenosine diphosphate P2Y12 receptor may prevent migraine through preventing platelet aggregation, vasoconstriction, and the fall of systemic blood pressure [30, 36]. What is more, a recent study found that clopidogrel suppressed nitroglycerin-induced microglial morphological changes (process retraction) and inducible nitric oxide synthase production in the trigeminal nucleus caudalis in chronic migraine mice via inhibiting the microglial P2Y12 receptor activity, which may be a mechanism of relieving migraine attacks, suggesting that both the microglia and platelet should be considered targets of the P2Y12 inhibitor [37].

The pooled analysis of the enrolled studies showed that P2Y12 inhibitor responders had an almost parallel MHA response to subsequent PFO closure after discontinuing the medication, suggesting that at least in these responsive populations, the right to left passage of venous platelet activation or aggregation acts as a main MHA mechanism/trigger. In the past several years, despite a marked reduction in MHA frequency following PFO closure having been reported in observational series [38, 39], randomized trials have not confirmed this [40, 41]. Since most migraineurs do not have a right to left shunt, alternate MHA mechanisms must exist. It is highly likely that some migraineurs have a PFO that is incidental to the MHA, and the inability to distinguish the PFOs that were mechanistically related to MHA from those that are simply incidental perhaps could explain why prior randomized PFO/MHA trials have failed [9]. P2Y12 inhibitor responsiveness may act as a screening tool to determine the suitability for PFO closure. Also as a common medication taken after the closure, it is important to consider a confounding factor of the P2Y12 inhibitor, which may lead to overestimating the real effect of shunt closure in future RCTs.

Change in preexisting MHA (exacerbation, improvement) or development of new-onset MHA is broadly acknowledged neurological alterations that can follow ASDC. Approximately 15% of patients had new-onset migraine attacks following transcatheter ASDC [7, 8]. The pathophysiology linking interatrial shunts and MHA is probably different from the one linking ASDC with new-onset migraine. In the enrolled RCT [14], the addition of clopidogrel therapy reduced the severity of migraine, with no patient in the dual antiplatelet therapy group presenting moderately or severely disabling headache compared with more than one-third of the patients receiving single aspirin therapy. The pooled lower rate of new-onset migraine after ASDC benefiting from the use of the P2Y12 inhibitor also indicates a platelet-involved mechanism. The occurrence of increased platelet aggregation following ASDC has been reported, which may have increased the release of serotonin [42]. Serotonin released from platelets which activated on the left atrial disc of a device may also be involved in the pathogenesis. Another possibility of mechanisms of MHA is the presence of cerebral embolism due to the formation of a thrombus on the surface of the atrial septal device.

With regard to the safety of the P2Y12 inhibitor, bleeding complications are the most considered. Overall, the most common minor bleeding event was cutaneous bruising in the present studies; major bleeding rarely occurred, except one gastrointestinal haemorrhage and one pelvic haematoma reported in one study in the aspirin plus clopidogrel group [15]. These findings suggest that the safety of the P2Y12 inhibitor is acceptable in the MHA/PFO population, especially if used as a short-term screening tool to determine the suitability for PFO closure.

Our meta-analysis has strengths. To the best of our knowledge, this is the first meta-analysis to evaluate the efficacy of the platelet P2Y12 inhibitor in the treatment of migraine, which correlates closely with clinical practice and helps at least specific subsets of migraineurs benefiting from this medication.

Limitations of our meta-analysis should also be addressed here. The major limitation of this review is that only two RCTs were investigated, other studies included were all observational and mostly retrospective designs with heterogeneous baseline characteristics. Due to the quality and a low number of trials included, we could not accurately and quantitatively describe the effectiveness of P2Y12 inhibitors on migraine in terms of headache intensity, duration, and frequency and also could not perform subgroup and sensitivity analysis. Thus, although we found that the P2Y12 inhibitor was efficacious in the prevention of migraine, considering the level of evidence, these results are far from robust.

## 5. Conclusions

Our meta-analysis showed that the P2Y12 platelet inhibitor may have a primary prophylactic role in migraine patients with or without PFO and preventing new-onset MHA after ASDC. Responsiveness of the P2Y12 inhibitor could help select candidates who would benefit from PFO closure. However, in view of the small and underpowered trials enrolled, these results are far from robust. This warrants further large-scale research to explore the role of the P2Y12 inhibitor, particularly in a proportion of migraine patients.

## Figures and Tables

**Table 1:** Demographic and clinical characteristics of included studies for migraine patients with or without PFO.

Study	Country	Design	Patients	Mean age (yr)	Treatments		Sample size	Dropouts	Efficacy results	Adverse events
					Intervention	Control				
Chambers et al. [9]	UK	Parallel RCT	Migraineurs with four to 15 headache days per 28-day month	45	Clopidogrel 75 mg/d for three months	Placebo once daily for three months	35 vs. 36	4 vs. 5	The number of headache days fell by 1.9 on clopidogrel and 1.8 on placebo (0.02, CI: -2.07 to 2.12)	Total: 17 vs. 14 Bruises: 5 vs. 3 Nosebleed: 3 vs. 1

Reisman et al. [10]	USA	Open-label single-arm feasibility trial	Patients ≥6 monthly MHA days with PFO	36.3	Ticagrelor 90 mg twice/d for 28 days	NA	40	2	17 (43%) participants were ticagrelor responders. No patient had complete relief	Total: 13 Bruises: 5

Sommer et al. [11]	USA	Open-label single-arm feasibility trial	Patients with MHA and PFO	37.9	Clopidogrel 75 mg/d for 1-3 months. Nonresponders were offered prasugrel	NA	136	1	90 (66%) patients were thienopyridines responders. 56 (70%) of clopidogrel had complete or near complete relief	Mild bleeding: 3 Cutaneous bruising: 20 Fatigue: <5%

Spencer et al. [12]	USA	Open-label single-arm feasibility trial	Patients with severe MHA and PFO	32.3	Clopidogrel 75 mg/d for 4 weeks	NA	15	1	13 (87%) patients were responders. 9 (60%) had complete relief	Allergic symptoms: 1

RCT: randomized controlled trial; NA: not applicable; PFO: patent foramen ovale; MHA: migraine headache.

**Table 2:** Demographic and clinical characteristics of included studies for new-onset MHA after transcatheter ASDC.

Study	Country	Design	Patients	Mean age (yr)	Treatments		Sample size	Dropouts	Efficacy results	Adverse events
					Intervention	Control				
Kato et al. [13]	Japan	Retrospective observational study	Patients underwent ASDC with or without migraine	27	Clopidogrel, ticlopidine, aspirin+ticlopidine, and ticlopidine+warfarin following ASDC for 6 months	Aspirin, asprin+warfarin, and dipyridamole+warfarin following ASDC for 6 months	50 vs. 157	NA	New-onset MHA occurred in 23 (11%) patients (4 vs. 19)	NA

Rodés-Cabau et al. [14]	Canada	Parallel RCT	Patients with an indication for ASDC and no history of migraine	49	Aspirin 80 mg/d+clopidogrel 75 mg/d following ASDC for 3 months	Aspirin 80 mg/d+placebo for 3 months	84 vs. 87	16 vs. 16	New-onset MHA occurred in 27 (16%) patients (8 vs. 19). Number of monthly migraine days: 0.4 vs. 1.4 days); difference: -1.02 (95% CI, -1.94 to -0.10)	Total: 14 vs. 19 Major bleeding: 0 Minor bleeding: 5 vs. 1

Wilmshurst et al. [15]	UK	Retrospective observational study	Patients underwent ASDC with or without migraine	39.6	Aspirin 150-300 mg/d for six months+clopidogrel 75 mg/d for the first month	Aspirin 150-300 mg/d for 6 months	90 vs. 71	NA	New-onset MHA occurred in 12 (7%) patients (3 vs. 9)	Major bleeding: 1 Gastrointestinal haemorrhage and 1 pelvic haematoma in aspirin+clopidogrel group

RCT: randomized controlled trial; NA: not applicable; ASDC: atrial septal defect closure; MHA: migraine headache.

## Data Availability

The data that support the findings of this study are available on request from the corresponding author.

## Supplementary Materials

Supplementary Materials Supplemental Table 1: modified Jadad score for included randomized controlled trials.
