# Supplementary material for: Platelet P2Y12 Inhibitor in the Treatment and Prevention of Migraine: A Systematic Review and Meta-Analysis
Source: Behav Neurol. 2022 Mar 20;2022:2118740. doi: 10.1155/2022/2118740 (PMC8958059; doi:10.1155/2022/2118740)
Supplement: Supplementary Materials — Supplemental Table 1: modified Jadad score for included randomized controlled trials. [file 2118740.f1.pdf]

Supplemental Table 1. Modified Jadad score for included randomized controlled trials

Study	Sequence generation	Randomization concealment	Blinding	Withdrawals and dropouts descriptions	Total Score*
Chambers et al [9]	2	2	2	1	7
Rodés-Cabau et al [14]	2	2	2	1	7

*Scores: 0-7 points; poor quality if ≤ 3
